# Human papillomavirus DNA positivity and seropositivity in rural Chinese men and women: a population-based cross-sectional study

**DOI:** 10.1038/srep26343

**Published:** 2016-05-23

**Authors:** Fangfang Liu, Qiuju Deng, Chanyuan Zhang, Yaqi Pan, Ying Liu, Zhonghu He, Min Sun, Mengfei Liu, Jingjing Li, Xiang Li, Chaoting Zhang, Dong Hang, Tao Ning, Chuanhai Guo, Yongmei Liang, Ruiping Xu, Lixin Zhang, Hong Cai, Yang Ke

**Affiliations:** 1 Key Laboratory of Carcinogenesis and Translational Research (Ministry of Education/Beijing), Laboratory of Genetics, Peking University Cancer Hospital & Institute, Beijing, China; 2 Anyang Cancer Hospital, Anyang, Henan Province, People’s Republic of China

## Abstract

Data on simultaneous analysis of human papillomavirus (HPV) DNA and serology and the correlations within a single general population are limited. Among 1603 men and 2187 women enrolled from rural China, serum antibodies against bacterially derived GST-L1 fusion proteins of HPV were assessed with multiplexed serology and HPV DNA was evaluated with PCR-based sequencing. Few subjects were dually positive to HPV DNA and serum antibodies for any HPV (6.6% of men and 3.1% of women). The proportion of men ever having been infected with any HPV (DNA and/or antibody positive) was higher than that of women (71.0% *vs.* 65.2%, *P* < 0.001). Type-specific association was observed for genital HPV infection and HPV seropositivity among women but not among men. A positive correlation between the number of lifetime sexual partners and positivity for oncogenic HPV DNA and/or antibodies was found in men but was absent in women. Among 762 couples, the presence of HPV DNA and/or antibodies in one partner was positively associated with the identical HPV type in the other partner. These findings may reflect a site-specific natural course of HPV infection and further understanding of the epidemiology of HPV.

Genital human papillomavirus (HPV) infection which is mainly sexually transmitted is an etiologic factor for malignant lesions of the cervix, vagina, vulva, and penis, and genital warts^1^. HPV DNA and serum antibodies are two markers commonly used for measuring HPV exposure. HPV DNA directly reflects current infection status, but most infections clear within 6–12 months^2^. Serological response indicates past HPV exposure, however not all natural HPV infections (~50% of cervical HPV infection) lead to seroconversion according to data collected using HPV Virus Like Particle (VLP) ELISA^3^,^4^, which is not as sensitive as Luminex approach. Both HPV DNA data and serology data, each with its particular advantages and disadvantages as a marker for HPV infection, should thus be used together to better evaluate HPV exposure. This approach may benefit characterization of HPV epidemiologic profiles, such as the prevalence and associated factors for ever having been infected with HPV (DNA positive and/or antibody positive), and thus help inform HPV prevention strategies (e.g. HPV vaccination).

However, until now much remains to be known about simultaneous status of HPV DNA and serology and its associated factors within given general populations worldwide, especially in men^5^. Additionally, available data regarding the correlation of genital HPV DNA detection and serologic response is limited. Previous cross-sectional studies with both DNA and serologic outcomes have been based largely on particular high-risk groups of females such as patients from sexually transmitted disease clinics^5^,^6^. For the most part these studies have been limited in sample size and HPV types evaluated, and evaluation has focused mainly on HPV-16. Moreover these studies have yielded a wide range of values for simultaneously negative HPV DNA and serology (62.1%-99.4% for HPV-16) and results regarding type-specific correlation of these two indices of HPV exposure have been inconsistent^5^,^6^.

Previously, we reported an overall seroprevalence of 64.8% for HPV-3, 6, 11, 16, 18, 45, 52, 57, 58, and/or 75 in 5548 healthy males and females aged 25–65 in rural China^7^. The aims of this population-based cross-sectional study are to evaluate the simultaneous positivity of both HPV DNA and serum antibodies, to explore the risk factors for ever having been infected with HPV, and to assess the correlations of HPV DNA status and antibody response based on this general population.

## Results

### Participant characteristics

Among 4996 eligible subjects, 3790 (75.9%) participants showed valid results for genital HPV DNA and serological outcomes. The chief reasons why the remaining 1206 individuals who were generally more likely to be younger men, were not included in the analysis, were either failure to respond due to employment outside of Anyang, or negativity for beta-globin. Among these 3790 subjects, the median age was 44 years (range, 25–65) and the male-female ratio was 0.7 (1603/2187). Most subjects had less than 9 years of education (91.7%) and were married or cohabiting with a partner (94.9%). More males habitually worked for long periods outside of their local areas of residency as compared to females (27.9% *vs.* 1.6%) (Table 1). Cigarette smoking and alcohol drinking were common among males (64.3%; 33.1%), but rare among females (0.2%; 0.4%). With respect to sexual behaviors, 11.2% men and 8.9% women reported sex debut at an earlier age (age ≤19). More males reported having ≥3 lifetime sexual partners than females (9.2% *vs.* 0.1%).

### HPV DNA positivity and seropositivity

Both overall and age-specific HPV seroprevalence was higher than DNA prevalence (Table 2, Supplementary Figure S1). In terms of combined HPV DNA and seroprevalence, while few men (any HPV: 6.6%; oncogenic HPV: 1.4%; non-oncogenic HPV: 2.7%) or women (any HPV: 3.1%; oncogenic HPV: 1.2%; non-oncogenic HPV: 0.8%) were simultaneously positive for both HPV DNA and antibodies, many more men (any HPV: 29.0%; oncogenic HPV: 65.4%; non-oncogenic HPV: 40.5%) and women (any HPV: 34.8%; oncogenic HPV: 68.8%; non-oncogenic HPV: 47.6%) were negative for both DNA and antibodies (Table 2). The proportion of men who were DNA positive and/or antibody positive for any HPV was significantly higher than that for women (71.0% *vs.* 65.2%, *P* < 0.001). The same pattern was seen for oncogenic HPV (34.6% *vs.* 31.2%, *P* = 0.026) and HPV-16 (13.0% *vs.* 11.9%, *P* < 0.001), which was the most prevalent type for both genders. Of the other four oncogenic HPV types evaluated, the proportions of HPV positivity ranged from 5.4% (HPV-52) to 10.2% (HPV-18; HPV-58) among men and ranged from 3.8% (HPV-52) to 11.0% (HPV-58) among women.

The percentage of HPV DNA positive men who were positive for HPV antibodies of the same oncogenic type was 11.3% among men and 14.3% among women (Data not shown). Regarding type-specific positivity for HPV antibodies of the same oncogenic type as the HPV DNA, the proportions were 0.0% for HPV-45, 5.6% for HPV-18, 8.9% for HPV-16, 27.3% for HPV-58 and 33.3% for HPV-52 among men. The proportions were 0.0% for HPV-18, 12.5% for both HPV-16 and HPV-52, 20.0% for HPV-58, and 25.0% for HPV-45 among women.

The pattern of the age-related HPV positivity (DNA positive and/or antibody positive) was driven mainly by HPV seroprevalence (Supplementary Figure S1). The oncogenic HPV positivity generally increased with age among women (*P*_*trend*_ = 0.017) and was characterized by a highest peak value at 56–60 years (36.8%). The age-stratified prevalence for oncogenic HPV was relatively constant with age among men except for a peak at 46–50 (42.5%).

### Association of HPV DNA positivity and seropositivity

In type-specific analysis among females, the presence of antibody against one specific HPV type in sera was associated with the presence of the same HPV type in a cervical specimen (Adjusted OR = 1.64, 95% CI: 1.02–2.62) (Table 3). With stratification by oncogenicity, these associations were also observed for both oncogenic HPV (Adjusted OR = 1.91, 95% CI: 1.01–3.60) and non-oncogenic HPV (Adjusted OR = 2.02, 95% CI: 0.95–4.26), although the latter did not achieve statistical significance (*P* = 0.066). However, among males, none of these correlations of genital HPV DNA positivity with seropositivity was found.

### Risk factors for HPV positivity (DNA positive and/or antibody positive)

Among females, increasing age (Adjusted *P*_trend_ = 0.017) and higher education levels (Adjusted *P*_trend_ = 0.008) were positively associated with oncogenic HPV positivity (Table 4). Among males, working outside of the local area conferred a greater risk of non-oncogenic HPV positivity as compared to farming or working locally (Adjusted OR = 1.30, 95% CI: 1.02–1.65). Men reporting more sexual partners had a significantly higher risk for oncogenic HPV positivity (Adjusted OR = 1.59, 95% CI: 1.11–2.28; ≥3 partners *vs.* 0–2 partners).

### Correlation of spousal HPV positivity (DNA positive and/or antibody positive)

Of the 762 couples where both partners had valid DNA and serologic data, the presence of HPV DNA and/or antibodies in one partner was positively associated with the identical HPV type in the other partner using type-specific analysis (Adjusted OR = 1.80, 95% CI: 1.51–2.13) (Table 5). With stratification by oncogenicity, spousal correlations as described above were also observed for both oncogenic HPV (Adjusted OR = 1.55, 95% CI: 1.09–2.20) and non-oncogenic HPV (Adjusted OR = 1.59, 95% CI: 1.30–1.94).

## Discussion

This is the first population-based study to investigate HPV DNA positivity and seropositivity together with correlations between these two HPV markers in both men and women within a single defined test population from rural China. In this study more subjects were simultaneously negative for both HPV DNA and serum antibodies than positive for both HPV DNA and antibodies. Type-specific association of genital HPV infection and HPV seropositivity was observed in women but not in men. The proportion of ever having been infected with HPV differed significantly with gender, sexual behavior and spousal infection status. These data improve our knowledge of the epidemiology and natural history of HPV infection.

In this study population, a very small percentage of individuals were positive for both HPV DNA and serum antibodies for any oncogenic HPV (1.4% of males and 1.2% of females). For HPV-16, which was most frequently evaluated previously, the proportion was even smaller (0.3% of males and 0.3% of females), in agreement with most studies^5^,^8^. Based on the results of clinical trials, this subset of exposed subjects would probably not benefit from receiving vaccines against the infected type(s)^9^,^10^. Regarding the type-specific concordance of HPV infection and antibody response, low percentage of HPV DNA positives who were also seropositive for the same type was observed. The weak concordance may be partly due to the usage of GST-L1 fusion proteins as antigens. Since GST-tag does not allow L1 proteins to spontaneously form VLP, it is possible that more non-specific binding of IgG antibodies from serum may occur as compared with the case for real virus particle or VLP or pseudovirion. Using mammalian derived HPV pseudovirions as antigens in multiplexed format, agreement for DNA and antibodies was seen by Faust^11^. Using GST-L1 as antigens, relatively weak concordance of natural infection and GST-L1 serology has been found by us and other groups, both for genital mucosal HPVs^12^ and for cutaneous HPVs^13^. Although GST-L1-based assay used in this study may result in less specificity to some extent, due to the ease of antigen production and purification as well as the comparable specificity, it has been proven to be an important tool for high-throughput analysis of multiple HPV type seroreactivity in large-scale epidemiological studies^14^,^15^,^16^.

Despite the rarity of concurrent detection of genital HPV DNA and serum antibodies and weak concordance of these two indices of HPV exposure, we observed that women who were HPV DNA positive were significantly more likely to be seropositive for antibodies against the same HPV type, as compared with HPV DNA negative women. However, no such DNA and antibody type-specific association was found in men. This gender difference may reflect a natural course of infection which is site-specific. Infection in the dry keratinized epithelium of the male genitalia may result less frequently in seroconversion than infection in the soft mucosal surfaces of the cervix^17^,^18^,^19^. Another explanation is that infection in men tends to be of shorter duration and lower viral load^20^, which may be less likely to elicit a humoral immune response^21^,^22^. Differences in other factors such as genetic and behavioral characteristics may also contribute to this gender disparity in seroconversion. Because of the transient nature of HPV infection, time lag in antibody development following infection, limited seroconversion rates, and gradual waning of the antibody response^4^, cross-sectional study design has inherent limitations in establishing dynamic correlations, and thus longitudinal studies with extensive follow-up are warranted to further investigate the association of HPV infection at different anatomical sites and the antibody response which is elicited.

Approximately 2/3 of this study population (65.4% of males and 68.8% of females) was simultaneously negative for oncogenic HPV DNA and antibody, which indicated that there was no evidence for current or past oncogenic HPV infection among this subset of subjects (Admittedly, serology measurements may underestimate cumulative exposure to HPV due to reasons such as low seroconversion). Data from clinical trials have demonstrated that HPV vaccination would be most effective at protecting against HPV-related cancer among these “unexposed” individuals^23^,^24^. The remaining individuals in the study, defined as ever having been infected with HPV, were DNA positive and/or antibody positive. HPV-16 was the most frequently detected oncogenic type, followed by HPV-18 and HPV-58, consistent with most previous reports regarding the spectrum of HPV infection in Asia^25^,^26^. This study showed HPV-16 positivity in 11.9% of females, which is in the middle of the prevalence range (0.6% to 27.0%) as reported in a global review of Asian females with a mean age of ~40 years^5^. The proportion of men who fell into the DNA positive and/or antibody positive category which has rarely been investigated in the general male population worldwide, was significantly higher than that of women (any HPV: 71.0% *vs.* 65.2%; oncogenic HPV: 34.6% *vs.* 31.2%; HPV-16: 13.0% *vs.* 11.9%) in this population. These gender-related disparities in HPV positivity are of uncertain cause. However, much higher genital HPV DNA prevalence (any HPV: 11.0% *vs.* 5.0%; oncogenic HPV: 5.0% *vs.* 3.5%; HPV-16: 2.8% *vs.* 2.2%) in combination with higher HPV seroprevalence (any HPV: 66.6% *vs.* 63.4%; oncogenic HPV: 31.0% *vs.* 28.9%; HPV-16: 10.4% *vs.* 9.6%) was observed in males as compared with females of this study population. This together with the fact that men have a lower likelihood of developing an immune response after natural HPV infection^27^,^28^, implies that more cumulative exposure of HPV may occur in men than in women in this rural Chinese population.

Regarding risk factors, the number of lifetime sexual partners in men was positively associated with oncogenic HPV positivity (DNA positive and/or antibody positive) as expected. However, no such association was found for non-oncogenic HPV, which may be due to insufficient statistical power because of underreporting of sexual partners. We observed that working outside the hometown area and away from the family, which is an indirect indicator of potential increased risk in sexual behaviors^29^, increased the risk of positivity for non-oncogenic HPV among men as compared to men working locally. However, none of the above associations was observed in females. These findings were similar to those in our previous study of HPV seroprevalence in the same population^7^. In western countries, relatively high numbers of lifetime sexual partners are both observed in men and women^30^,^31^. However, in rural China, sexual behavior is generally much more conservative, especially in women^32^. Fewer women than men reported having multiple lifetime sexual partners (≥2 partners: 2.3% *vs.* 15.5%, *P* < 0.001; ≥3 partners: 0.1% *vs.* 9.2%, *P* < 0.001). Thus, in addition to potential underreporting of sexual partners, conservative sexual behavior may partly explain the lack of statistical association of HPV positivity and sexual behavior indicators in females. Taken together, sexual practices may play a more important role in HPV exposure among men than among women in rural China.

Evaluating spousal concordance for HPV positivity, our results confirmed that in a large population-based sample HPV positivity in one partner increased the risk of type-specific HPV positivity in the other partner, in line with findings of spousal concordance analysis at other levels including HPV DNA level and sero-status level^7^,^32^. This result indicates HPV transmission may occur in couples. According to a cohort study of genital HPV transmission in couples conducted in the same population which was analyzed at HPV DNA level^32^, the risk of male-to-female transmission was much higher than that of female-to-male transmission for oncogenic HPV in couples. HPV transmission dynamics needs to be further explored in a larger study with more cycles of evaluation of both HPV DNA and seroreactivity.

Several limitations of this study must be noted. First, the nonparticipation and exclusion of beta-globin negative participants may undermine the generalizability of study results. Second, underreporting of risky sexual behaviors resulting from concern about the potential for social stigma may dilute their true effect on HPV positivity. Finally, in order to more accurately assess the prevalence of HPV exposure and the association of HPV DNA positivity and seropositivity, longitudinal data regarding HPV infection and antibody response both in adults and youngsters would be more informative.

In summary, the higher proportion of HPV DNA and antibody positivity in men as compared to women, and differing patterns of HPV DNA and antibody association across gender in rural China suggest a site-specific natural course of infection. These findings together with the risk factors and spousal correlations for ever having been infected with HPV provide a basis for further understanding the epidemiological profiles of HPV infection and developing HPV prevention programs.

## Materials and Methods

### Study population

A population-based esophageal cancer cohort study was initiated in rural Anyang, China in 2007–2009^33^. The current investigation was conducted in 6 of the 9 villages which were cluster-sampled to establish the baseline of this cohort. Eligibility criteria were as follows: 1) permanent residency in selected villages; 2) age 25–65; 3) no prior diagnosis of cancer, disease involving immunodeficiency, or cardiovascular illness; 4) no prior history of infection with HBV, HCV, or HIV; and 5) willingness to participate. As prophylactic HPV vaccines are still under clinical evaluation and have not been used in China, all participants were unvaccinated. All participants provided written informed consent, and the study was approved by the Institutional Review Board of the School of Oncology, Peking University. The methods were carried out in accordance with the approved guidelines.

### Specimen and data collection

As previously described^32^,^33^,^34^, exfoliated genital cells were collected by an experienced clinician using saline-moistened cotton swabs. Briefly, for males, exfoliated cells were collected from the penile shaft, glans penis, coronal sulcus, and scrotum by swabbing five times at each site. For females, cervical exfoliated cells were collected by insertion of a swab into the cervix with 360° rotation five times. The cells collected on the swab were then rinsed in a vial containing 0.9% saline solution. All vials were centrifuged at 3000 g for 10 min and the supernatants were decanted. Sera were also collected from each participant for antibody testing. Specimens were stored at −70 °C pending evaluation.

Computer-aided interviews were administered in private rooms by interviewers of the same gender as the participant. Information on demographic characteristics, cigarette consumption (defined as at least 1 cigarette per day for 1 year or more), alcohol consumption (defined as use of Chinese liquor at least twice per week for 1 year or more), and characteristics of sexual behavior was collected during these interviews. Information on spouses was also obtained to identify couples among the study subjects, which provided us an opportunity to examine correlation of HPV positivity within couples.

### HPV DNA detection and typing

DNA was purified on a Biomek 3000 automated workstation using the E.Z.N.A.TM Mag-Bind Tissue DNA Kit (Omega Bio-Tek, Inc.). The human β-globin gene was evaluated in all extracted and purified DNA samples. For β-globin positive samples, PCR was used to evaluate for HPV DNA using a highly sensitive primer set (SPF1/GP6+)^35^,^36^. PCR products which consisted of amplified 184-bp fragments of the L1 gene from HPV positive specimens were purified and subsequently directly sequenced on an ABI 3730XL DNA Analyzer to determine specific HPV types^35^. A broad spectrum of HPV types was detectable using this SPF1/GP6+-mediated PCR and sequencing approach (Oncogenic: HPV-16, 18, 26, 31, 35, 39, 45, 51, 52, 56, 58, 59, 66, and 68; non-oncogenic: HPV-2, 3, 6, 7, 10, 11, 27, 29, 30, 32, 33, 37, 40, 42, 43, 44, 53, 54, 55, 57, 61, 62, 67, 69, 70, 72, 73, 74, 75, 77, 81, 82, 83, 84, 87, 89, 90, 91, 94, and 127)^35^,^36^,^37^. Rigorous quality control procedures were conducted throughout the study to avoid contamination^38^,^39^.

### Multiplex serology

The expression and preparation of recombinant proteins, and the multiplex serologic assay developed by Waterboer *et al*. has been previously described in detail^7^,^15^. Briefly, glutathione S-transferase (GST)-L1-FLAG fusion proteins of each HPV type (the ten most locally prevalent HPV DNA types included HPV-3, 6, 11, 16, 18, 45, 52, 57, 58, and 75) from cleared lysates were affinity-purified in one step through binding to glutathione casein-coated fluorescence-labeled beads. Each antigen was bound to a bead set of a different color. All of these fusion protein-loaded bead sets were mixed. Sera were pre-incubated in PBS at 1:50 containing 1 mg/mL casein, 2 mg/mL lysate from bacteria expressing GST-FLAG tag alone, 0.5% polyvinylalcohol (PVA, Sigma-Aldrich), 0.8% polyvinylpyrrolidone (PVP, Sigma-Aldrich) and 2.5% Super chemiblock (Millipore, Billerica, MA, USA). Serum dilutions were incubated with an equal volume of mixed bead sets, resulting in a final dilution of 1:100. Bound antibodies were evaluated with biotin labeled goat-anti human IgG (H+L) (KPL, Gaithersburg, MD) and streptavidin-R-phycoerythrin (Invitrogen). A Bio-Plex 200 system (BIO-RAD, USA) was used to count the beads and quantify the fluorescence. The median fluorescence intensity (MFI) of beads coupled with GST only was used to calculate the background fluorescence level.

Sera from quadrivalent HPV (types 6, 11, 16, 18) vaccine recipients were pooled and included as the positive standard on each plate every day. The inter-plate coefficients of variation (CVs) for HPV-16 L1 across all days ranged from 2.0 to 24.0%, with a median of 15.2%. Reproducibility of measurements for serologic response to HPV-6 and 16 L1 was assessed by randomly selecting 419 sera for blind replicate tests. The correlation coefficients for the MFI values from two assays were 0.78 (HPV-6) and 0.87 (HPV-16).

Seropositivity thresholds of MFI levels for each mucosal HPV type (HPV-6, 11, 16, 18, 45, 52, and 58) were defined using data from 123 Chinese female students (age 18–24) who reported no history of sexual intercourse. The cut-off was set at three standard deviations above the mean level of MFI reactivity among the remaining samples after an iterative process to exclude positive outliers^40^. A cut-off of 200 MFI was arbitrarily set to define seropositivity for cutaneous HPV (HPV-3, 57, and 75)^16^,^41^.

### Statistical analysis

Ten HPV types (HPV-3, 6, 11, 16, 18, 45, 52, 57, 58, and 75) evaluated for both HPV DNA positivity and seropositivity were included for analysis. A sample was considered positive for any HPV if at least one of these 10 HPV types was detected. Groups of oncogenic (HPV-16, 18, 45, 52, and 58) and non-oncogenic types (HPV-3, 6, 11, 57, and 75) were classified according to previous reports^35^,^37^. The comparison group for all analysis regarding each HPV risk group included subjects without the corresponding types, regardless of the presence or absence of other types. For instance, for analysis of oncogenic HPV, subjects with HPV-16, 18, 45, 52, and/or 58 were included as a risk group, and subjects without any of these types (even if they showed presence of HPV-3, 6, 11, 57, and/or 75) were classified as a reference group. Unless otherwise specified, HPV positivity, ever having been infected with HPV, in this study is defined as positivity for HPV DNA and/or antibodies (That is, except for individuals with simultaneously negative HPV DNA and serology, all subjects belonged to the HPV positive group).

Multivariate logistic regression models were used to examine the association of HPV DNA positivity and seropositivity. The primary exposure variable was the presence in the genitalia of HPV infection of a particular type, and the outcome variable was the presence of serum antibody of the same HPV type. Three sets of models were considered: 1) positivity for any HPV type in the list above in this statistical analysis section; 2) positivity for any oncogenic HPV type in the list; 3) positivity for any non-oncogenic HPV type in the list. Because each participant contributed more than one observation (there is one HPV outcome for each HPV type) in each set of models, generalized estimating equations (GEEs) with an exchangeable correlation structure were applied to adjust for possible correlations of different HPV types (analysis at infection-level). To assess the type-specific association of HPV positivity (positive for HPV DNA and/or antibodies) between spouses, the same method of analysis at infection-level was used. The primary exposure variable was the presence of genital HPV DNA or serum antibody of a particular type in the male partner in a couple. The outcome variable was the presence of genital HPV DNA or serum antibody of the same HPV type in his spouse.

To identify independent determinants of HPV positivity, variables that were statistically significant in univariate analyses, together with factors previously reported to be potentially associated were entered into multivariate logistic regression models (analysis at person-level). Trend tests were conducted across ordered groups treating categorical variables as continuous covariates.

All statistical analysis was performed using Stata for Windows (version 11.2, StataCorp, College Station, TX). *P* values less than 0.05 (two-sided) were considered to be statistically significant.

## Additional Information

**How to cite this article**: Liu, F. *et al*. Human papillomavirus DNA positivity and seropositivity in rural Chinese men and women: a population-based cross-sectional study. *Sci. Rep.* **6**, 26343; doi: 10.1038/srep26343 (2016).

## Supplementary Material

Supplementary Information

## Figures and Tables

**Table 1:** Selected demographic and behavior-related characteristics for 1603 men and 2187 women included in the study of population-based HPV DNA positivity and seropositivity in rural Anyang, China, 2007–2009.

**Variables**	**Male**	**Female**
Total	1603 (100.0)	2187 (100.0)
Age, years		
25–35	475 (29.6)	472 (21.6)
36–45	574 (35.8)	796 (36.4)
46–55	296 (18.5)	468 (21.4)
56–65	258 (16.1)	451 (20.6)
Education level		
Illiteracy, <1 year	57 (3.6)	510 (23.3)
Primary school, 1–6 years	432 (27.0)	835 (38.2)
Junior high school, 7–9 years	912 (56.9)	728 (33.3)
Senior high school or above, >9 years	202 (12.6)	114 (5.2)
Marital status		
Married or cohabiting	1503 (93.8)	2092 (95.7)
Not married, divorced, separated or widowed	100 (6.2)	95 (4.3)
Type of employment		
Farming or working in local area	1124 (70.1))	2053 (93.9)
Working outside local area	447 (27.9)	35 (1.6)
Other	32 (2.0)	99 (4.5)
Cigarette smoking		
Never	572 (35.7)	2182 (99.8)
Ever	1031 (64.3)	5 (0.2)
Alcohol consumption		
Never	1072 (66.9)	2178 (99.6)
Ever	531 (33.1)	9 (0.4)
Age at sex debut, years		
≤19	179 (11.2)	195 (8.9)
>19	1424 (88.8)	1992 (91.1)
Lifetime number of sexual partners		
0–2	1455 (90.8)	2184 (99.9)
≥3	148 (9.2)	3 (0.1)

Abbreviations: HPV, human papillomavirus.

**Table 2:** Concordance of genital HPV DNA and antibody positivity in paired genital specimens and sera from individual participants stratified by gender in rural Anyang, 2007–2009.

**Gender**	**HPV type**	**DNA+**	**Antibody+**	**DNA+ and antibody+**	**DNA+ and antibody-**	**DNA- and antibody+**	**DNA+ and/or antibody+**	**DNA- and antibody-**
		**No. (%)**						
Male (n = 1603)								
	Any HPV (n = 10)	176 (11.0)	1068 (66.6)	106 (6.6)	70 (4.4)	962 (60.0)	1138 (71.0)	465 (29.0)
	Oncogenic HPV (n = 5)	80 (5.0)	497 (31.0)	22 (1.4)	58 (3.6)	475 (29.6)	555 (34.6)	1048 (65.4)
	HPV-16	45 (2.8)	167 (10.4)	4 (0.3)	41 (2.6)	163 (10.2)	208 (13.0)	1395 (87.0)
	HPV-18	18 (1.1)	146 (9.1)	1 (0.1)	17 (1.1)	145 (9.1)	163 (10.2)	1440 (89.8)
	HPV-45	3 (0.2)	104 (6.5)	0 (0.0)	3 (0.2)	104 (6.5)	107 (6.7)	1496 (93.3)
	HPV-52	3 (0.2)	84 (5.2)	1 (0.1)	2 (0.1)	83 (5.2)	86 (5.4)	1517 (94.6)
	HPV-58	11 (0.7)	155 (9.7)	3 (0.2)	8 (0.5)	152 (9.5)	163 (10.2)	1440 (89.8)
	Non-oncogenic HPV (n = 5)	96 (6.0)	902 (56.3)	44 (2.7)	52 (3.2)	858 (53.5)	954 (59.5)	649 (40.5)
	HPV-3	49 (3.1)	353 (22.0)	12 (0.8)	37 (2.3)	341 (21.2)	390 (24.3)	1213 (75.7)
	HPV-6	4 (0.3)	368 (23.0)	1 (0.1)	3 (0.2)	367 (22.9)	371 (23.1)	1232 (76.9)
	HPV-11	12 (0.8)	227 (14.2)	1 (0.1)	11 (0.7)	226 (14.1)	238 (14.8)	1365 (85.2)
	HPV-57	22 (1.4)	154 (9.6)	4 (0.3)	18 (1.1)	150 (9.4)	172 (10.7)	1431 (89.3)
	HPV-75	9 (0.6)	355 (22.1)	1 (0.1)	8 (0.5)	354 (22.1)	363 (22.6)	1240 (77.4)
Female (n = 2187)								
	Any HPV (n = 10)	109 (5.0)	1386 (63.4)	68 (3.1)	41 (1.9)	1318 (60.3)	1427 (65.2)	760 (34.8)
	Oncogenic HPV (n = 5)	77 (3.5)	631 (28.9)	26 (1.2)	51 (2.3)	605 (27.7)	682 (31.2)	1505 (68.8)
	HPV-16	48 (2.2)	210 (9.6)	6 (0.3)	42 (1.9)	212 (9.2)	260 (11.9)	1935 (88.5)
	HPV-18	2 (0.1)	192 (8.8)	0 (0.0)	2 (0.1)	192 (8.8)	194 (8.9)	1993 (91.1)
	HPV-45	4 (0.2)	155 (7.1)	1 (0.1)	3 (0.1)	154 (7.0)	158 (7.2)	2029 (92.8)
	HPV-52	8 (0.4)	76 (3.5)	1 (0.1)	7 (0.3)	75 (3.4)	83 (3.8)	2104 (96.2)
	HPV-58	15 (0.7)	229 (10.5)	3 (0.1)	12 (0.6)	226 (10.3)	241 (11.0)	1946 (89.0)
	Non-oncogenic HPV (n = 5)	32 (1.5)	1133 (51.8)	19 (0.8)	13 (0.6)	1114 (50.9)	1146 (52.4)	1041 (47.6)
	HPV-3	26 (1.2)	440 (20.1)	8 (0.4)	18 (0.8)	432 (19.8)	458 (20.9)	1729 (79.1)
	HPV-6	2 (0.1)	427 (19.5)	1 (0.1)	1 (0.1)	426 (19.5)	428 (19.6)	1759 (80.4)
	HPV-11	0 (0.0)	316 (14.5)	0 (0.0)	0 (0.0)	316 (14.5)	316 (14.5)	1871 (85.6)
	HPV-57	4 (0.2)	207 (9.5)	1 (0.1)	3 (0.1)	206 (9.4)	210 (9.6)	1977 (90.4)
	HPV-75	0 (0.0)	484 (22.1)	0 (0.0)	0 (0.0)	484 (22.1)	484 (22.1)	1703 (77.9)

“DNA+” denotes positive for HPV DNA; “DNA-” denotes negative for HPV DNA; “antibody+” denotes positive for HPV antibody; “antibody-” denotes negative for HPV antibody.

Abbreviation: HPV, human papillomavirus.

**Table 3:** Type-specific association of genital HPV infection and HPV antibody response in paired genital specimens and sera from individual participants stratified by gender in rural Anyang, 2007–2009.

**Gender**	**HPV type**^a^	**HPV DNA status of external genitalia**	**HPV sero-status**		**Total**	**Crude OR (95% CI)**^b^	**Adjusted OR (95% CI)**^c^
			**Negative**	**Positive**			
Male (n = 1603)^d^							
	Any HPV(n = 10)	Negative	13769	148	13917	1.00	1.00
		Positive	2085	28	2113	1.28 (0.86–1.91)	1.28 (0.86–1.90)
		Total	15854	176	16030		
	Oncogenic HPV(n = 5)	Negative	7288	71	7359	1.00	1.00
		Positive	647	9	656	1.44 (0.72–2.87)	1.41 (0.71–2.81)
		Total	7935	80	8015		
	Non-oncogenic HPV(n = 5)	Negative	6481	77	6558	1.00	1.00
		Positive	1438	19	1457	1.18 (0.72–1.93)	1.18 (0.72–1.92)
		Total	7919	96	8015		
Female (n = 2187)^e^							
	Any HPV(n = 10)	Negative	19046	88	19134	1.00	1.00
		Positive	2715	21	2736	1.63 (1.02–2.61)	1.64 (1.02–2.62)
		Total	21761	109	21870		
	Oncogenic HPV(n = 5)	Negative	10007	66	10073	1.00	1.00
		Positive	851	11	862	1.89 (1.00–3.57)	1.91 (1.01–3.60)
		Total	10858	77	10935		
	Non-oncogenic HPV(n = 5)	Negative	9039	22	9061	1.00	1.00
		Positive	1864	10	1874	2.00 (0.95–4.22)	2.02 (0.95–4.26)
		Total	10903	32	10935		

Abbreviations: HPV, human papillomavirus; OR, odds ratio; CI, confidence interval.

^a^Groups of oncogenic (HPV-16, 18, 45, 52, and 58) and non-oncogenic types (HPV-3, 6, 11, 57, and 75) were classified according to previous reports.

^b^Crude ORs and 95% CIs were calculated by univariate logistic regression analyses with generalized estimating equations (GEE).

^c^Adjusted ORs and 95% CIs were calculated by multivariate logistic regression analyses with GEE including age, cigarette smoking, alcohol consumption, and lifetime number of sexual partners.

^d^Number of observations for type-specific analysis = 10 (total number of types detected) × 1603 (number of men). Stratified by oncogenicity, 8015 observations (5 × 1603) and 8015 observations (5 × 1603) were involved in type-specific analysis for oncogenic HPV and non-oncogenic HPV.

^e^Number of observations for type-specific analysis = 10 (total number of types detected) × 2187 (number of women). Stratified by oncogenicity, 10935 observations (5 × 2187) and 10935 observations (5 × 2187) were involved in type-specific analysis for oncogenic HPV and non-oncogenic HPV.

**Table 4:** Factors associated with HPV positivity (DNA positive and/or antibody positive) in 1603 men and 2187 women from rural Anyang, China, 2007–2009.

**Variables**	**Male (n = 1603)**						**Female (n = 2187)**					
	**Any HPV (n = 10)**^a^		**Oncogenic HPV (n = 5)**^a^		**Non-oncogenic HPV (n = 5)**^a^		**Any HPV (n = 10)**^a^		**Oncogenic HPV (n = 5)**^a^		**Non-oncogenic HPV (n = 5)**^a^	
	**%, Adjusted OR (95% CI)**^b^						**%, Adjusted OR (95% CI)**^b^					
Total	71.0		34.6		59.5		65.3		31.2		52.4	
Age, years												
25–35	72.8	1.00	35.8	1.00	62.3	1.00	65.3	1.00	30.5	1.00	53.4	1.00
36–45	68.8	0.84 (0.64–1.11)	32.2	0.87 (0.67–1.13)	56.3	0.81 (0.63–1.04)	62.8	0.91 (0.71–1.15)	28.9	0.94 (0.73–1.21)	50.4	0.89 (0.71–1.12)
46–55	73.3	1.08 (0.77–1.51)	36.8	1.10 (0.80–1.51)	62.2	1.05 (0.77–1.44)	65.2	1.07 (0.80–1.43)	31.4	1.15 (0.86–1.55)	51.9	1.01 (0.76–1.33)
56–65	69.8	0.90 (0.62–1.30)	35.3	1.02 (0.72–1.45)	58.5	0.98 (0.69–1.38)	69.6	1.30 (0.94–1.80)	35.7	1.43 (1.04–1.97)	55.4	1.18 (0.87–1.60)
*P* value for trend^c^		0.890		0.648		0.820		0.082		0.017		0.230
Education level												
Illiteracy, <1 year	70.2	1.00	33.3	1.00	57.9	1.00	64.7	1.00	30.2	1.00	51.0	1.00
Primary school, 1–6 years	70.6	1.05 (0.56–1.95)	35.7	1.20 (0.66–2.18)	56.9	0.99 (0.56–1.76)	65.8	1.22 (0.94–1.58)	30.9	1.25 (0.96–1.63)	53.4	1.21 (0.94–1.55)
Junior high school, 7–9 years	71.1	1.05 (0.57–1.95)	34.4	1.13 (0.62–2.05)	60.2	1.16 (0.65–2.05)	64.8	1.23 (0.93–1.64)	31.3	1.39 (1.03–1.86)	52.2	1.18 (0.90–1.55)
Senior high school or above, >9 years	71.8	1.05 (0.54–2.07)	33.7	1.03 (0.54–1.99)	62.4	1.31 (0.70–2.45)	66.7	1.30 (0.83–2.05)	36.8	1.76 (1.12–2.77)	52.6	1.15 (0.75–1.77)
*P* value for trend^c^		0.919		0.638		0.115		0.183		0.008		0.397
Marital status												
Married or cohabiting	70.9	1.00	34.2	1.00	59.4	1.00	65.1	1.00	31.0	1.00	52.4	1.00
Not married, divorced, separated or widowed	73.0	1.05 (0.66–1.69)	41.0	1.34 (0.87–2.06)	62.0	1.04 (0.67–1.59)	69.5	1.09 (0.69–1.75)	35.8	1.15 (0.73–1.81)	52.6	0.95 (0.61–1.46)
Type of employment												
Farming or working in local area	70.5	1.00	34.5	1.00	57.7	1.00	65.6	1.00	31.8	1.00	52.6	1.00
Working outside local area	71.8	1.04 (0.81–1.34)	34.5	1.00 (0.78–1.28)	64.0	1.30 (1.02–1.65)	48.6	0.51 (0.26–1.00)	20.0	0.55 (0.24–1.28)	40.0	0.61 (0.31–1.20)
Other	78.1	1.47 (0.62–3.49)	40.6	1.34 (0.64–2.79)	59.4	1.00 (0.48–2.07)	63.6	0.90 (0.59–1.38)	23.2	0.63 (0.39–1.02)	53.5	1.02 (0.68–1.53)
Cigarette smoking												
Never	70.6	1.00	36.2	1.00	58.0	1.00	65.2	1.00	31.3		52.3	1.00
Ever	71.2	1.03 (0.82–1.30)	33.8	0.86 (0.69–1.07)	60.3	1.12 (0.90–1.39)	80.0	1.75 (0.19–15.89)	0.0	—	80.0	3.36 (0.37–30.45)
Alcohol consumption												
Never	71.6	1.00	33.8	1.00	61.0	1.00	65.2	1.00	31.2	1.00	52.3	1.00
Ever	69.9	0.91 (0.72–1.15)	36.4	1.14 (0.91–1.42)	56.5	0.83 (0.67–1.03)	66.7	1.01 (0.25–4.08)	33.3	0.98 (0.24–4.00)	66.7	1.83 (0.45–7.40)
Age at sex debut, years												
≤19	73.7	1.00	34.1	1.00	63.1	1.00	68.7	1.00	35.4	1.00	52.3	1.00
>19	70.7	0.90 (0.62–1.30)	34.7	1.17 (0.83–1.66)	59.1	0.84 (0.60–1.17)	64.9	0.90 (0.65–1.26)	30.8	0.84 (0.61–1.16)	52.4	1.05 (0.77–1.44)
Lifetime number of sexual partners												
0–2	70.5	1.00	33.7	1.00	59.6	1.00	65.3	1.00	31.2	1.00	52.4	1.00
≥3	75.7	1.26 (0.84–1.89)	43.9	1.59 (1.11–2.28)	58.8	0.91 (0.63–1.30)	66.7	1.02 (0.09–11.32)	33.3	1.02 (0.09–11.34)	33.3	0.45 (0.04–5.02)

Abbreviations: HPV, human papillomavirus; OR, odds ratio; CI, confidence interval.

^a^Groups of oncogenic (HPV-16, 18, 45, 52, and 58) and non-oncogenic types (HPV-3, 6, 11, 57, and 75) were classified according to previous reports.

^b^OR and 95% CI were derived by multivariate logistic regression models which included all listed variables.

^c^*P* values for trend were derived by logistic regression analysis taking categorical variables as continuous variables.

**Table 5:** Type-specific spousal association of HPV positivity (DNA positive and/or antibody positive) in 762 heterosexual couples in rural Anyang, 2007–2009.

**HPV type**^a^	**HPV status of male partner**	**HPV status of female partner**^b^		**Total**	**Crude OR (95% CI)**^c^	**Adjusted OR (95% CI)**^d^
		**Negative**	**Positive**			
Any HPV (n = 10)						
	Negative	5795	771	6566	1.00	1.00
	Positive	851	203	1054	1.80 (1.52–2.14)	1.80 (1.51–2.13)
	Total	6646	974	7620		
Oncogenic HPV (n = 5)						
	Negative	3189	272	3461	1.00	1.00
	Positive	307	42	349	1.56 (1.10–2.21)	1.55 (1.09–2.20)
	Total	3496	314	3810		
Non-oncogenic HPV (n = 5)						
	Negative	2606	499	3105	1.00	1.00
	Positive	544	161	705	1.60 (1.31–1.95)	1.59 (1.30–1.94)
	Total	3150	660	3810		

Abbreviations: HPV, human papillomavirus; OR, odds ratio; CI, confidence interval.

^a^Groups of oncogenic (HPV-16, 18, 45, 52, and 58) and non-oncogenic types (HPV-3, 6, 11, 57, and 75) were classified according to previous reports. Number of observations for type-specific analysis = 10 (total number of types detected) × 762 (number of couples). Stratified by oncogenicity, 3810 observations (5 × 762) and 3810 observations (5 × 762) were involved in type-specific analysis for oncogenic HPV and non-oncogenic HPV.

^b^Any HPV status of female partners was analyzed corresponding to any HPV status of male partners. Parallel analyses were conducted for other HPV risk groups.

^c^Crude ORs and 95% CIs were calculated by univariate logistic regression analyses with generalized estimating equations (GEE).

^d^Adjusted ORs and 95% CIs were calculated by multivariate logistic regression analyses with GEE including age, cigarette smoking, alcohol consumption, and lifetime number of sexual partners.
