# Supplementary material for: Human papillomavirus DNA positivity and seropositivity in rural Chinese men and women: a population-based cross-sectional study
Source: Sci Rep. 2016 May 23;6:26343. doi: 10.1038/srep26343 (PMC4876329; doi:10.1038/srep26343)
Supplement: Supplementary Information [file srep26343-s1.doc]

**Human papillomavirus DNA positivity and seropositivity in rural Chinese men and women: a population-based cross-sectional study**

Fangfang Liu, Qiuju Deng, Chanyuan Zhang, Yaqi Pan, Ying Liu, Zhonghu He, Min Sun, Mengfei Liu, Jingjing Li, Xiang Li, Chaoting Zhang, Dong Hang, Tao Ning, Chuanhai Guo^1^, Yongmei Liang, Ruiping Xu, Lixin Zhang, Hong Cai and Yang Ke

**Supplementary Figure S1. Age-specific prevalence of HPV DNA, antibody and combinations (DNA positive and/or antibody positive) for 10 HPV types in healthy men and women from rural Anyang, China, 2007-2009.**

**Abbreviations:** HPV, human papillomavirus. A total of 10 human papillomavirus (HPV) types including 5 oncogenic HPV (HPV-16, 18, 45, 52, and 58) and 5 non-oncogenic HPV (HPV-3, 6, 11, 57, and 75) were analyzed in 1603 males and 2187 females.
